# *Cynomorium songaricum* Rupr. flavonoids improve cyclophosphamide-induced reproductive function damage by regulating the testosterone synthesis pathway

**DOI:** 10.3389/fphar.2024.1457780

**Published:** 2024-08-22

**Authors:** Jiarong Chen, Xiaoyue Yang, Zhongmei He, Weijia Chen, Yan Zhao, Jianming Li, Ying Zong, Rui Du

**Affiliations:** ^1^ College of Chinese Medicinal Materials, Jilin Agricultural University, Changchun, China; ^2^ Jilin Provincial Engineering Research Center for Efficient Breeding and Product Development of Sika Deer, Jilin Agricultural University, Changchun, China; ^3^ Key Laboratory of Animal Production and Product Quality and Safety, Ministry of Education, Jilin Agricultural University, Changchun, China

**Keywords:** *Cynomorium songaricum* Rupr., total flavonoids, reproductive function damage, testosterone synthesis pathway, testosterone (androgen)

## Abstract

**Introduction:**

The prevalence of male infertility has been increasing globally, necessitating the search for safe and nontoxic active compounds to alleviate reproductive dysfunction. Although the precise mechanism remains unknown, *Cynomorium songaricum* Rupr. (CS) extract has protective effects on the reproductive system. The effect of C. songaricum Rupr. flavonoids (CSF) on reproductive injury and testicular mesenchymal stem cell viability in male mice and TM3 cells was investigated.

**Methods:**

We explored the possible association between these effects and the testosterone (T) synthesis pathway. Mice were administered cyclophosphamide to induce reproductive damage, followed by CSF administration. Body mass and organ index were recorded. Pathological changes in T and the epididymis were observed using hematoxylin-eosin staining. ELISA measured the serum levels of T, luteinizing hormone (LH), gonadotropin-releasing hormone (GnRH), follicle-stimulating hormone (FSH), and estradiol (E_2_) in mice. Fructose and zinc ion levels in the seminal plasma were measured. TM3 cells were treated with Bisphenol A (BPA) and different concentrations of CSF, followed by proliferative evaluations using the CCK-8 assay and T and LH level assessments using ELISA. Furthermore, the expression of steroidogenic enzyme genes and proteins was investigated using western blotting and RT-PCR.

**Results:**

CSF exhibited a notable reduction in reproductive damage and improved pathological changes in testicular and epididymal tissues. CSF group demonstrated substantially higher levels of seminal plasma fructose and zinc ions; markedly elevated serum levels of T, LH, GnRH, and FSH; and lower levels of E2 than those of the model group. Intracellular T content and secretion of T and LH increase with CSF while effectively mitigating BPA-induced damage to TM3 cells. CSF group exhibited substantially higher gene and protein expression of steroidogenic enzymes than those of the model group, both *in vivo* and *in vitro*. CSF ameliorates reproductive impairment by enhancing the expression of pivotal enzymes involved in synthesizing T.

**Discussion:**

CSF ameliorates cyclophosphamide-induced reproductive impairment and bisphenol A-induced TM3 cell damage in mice by regulating sex hormone levels in the Hypothalamic-Pituitary-Gonadal Axis (HPG axis) and upregulating the expression of steroidogenic enzymes. Therefore, CS is a potential treatment for male reproductive impairment.

## Introduction

Male infertility is becoming increasingly prevalent worldwide. Potential causes include environmental factors, endocrine abnormalities, immune system dysfunction, and genetic factors (Li et al., 2020; Kirsten et al., 2020). Approximately 46% of male patients with infertility are affected by oligospermia, which is characterized by reduced sperm count and viability (Zhang et al., 2020). Research indicates that in China, sperm quality declines by 1% annually, whereas Western countries experience a decline of 2.6% in sperm density and 0.3% in sperm viability rates per year (Chyra-Jach et al., 2018; Yang et al., 2020). In light of the growing demand for fertility and the escalating incidence of infertility, it is imperative to explore safe and non-toxic active compounds for the treatment of reproductive impairment.

Testosterone, a vital hormone responsible for male secondary sexual characteristics, is primarily secreted by testicular interstitial cells, with 95% of its production regulated by the HPG axis (Wang et al., 2022). Within this axis, the hypothalamus produces GnRH, which stimulates the pituitary gland to secrete FSH and LH. These hormones stimulate the testes, initiate spermatogenesis, and trigger T secretion. The feedback loop is completed as T acts on the hypothalamus and pituitary gland through negative feedback, regulating endocrine hormones and maintaining normal function (Zhang et al., 2021). Several studies have shown that oxidative stress may contribute to male infertility, and increased levels of ROS can affect both spermatogenesis and the HPG axis (Shokoohi et al., 2022).

The steroidogenic enzyme pathway is the main pathway for T synthesis. StAR is a key factor in testicular interstitial cells that promotes cholesterol transport from the outer mitochondrial membrane to the inner membrane. Cholesterol passes through the outer mitochondrial membrane into the inner mitochondrial membrane under the regulation of STAR and is cleaved by CYP11A1 to pregnenolone, which is converted into progesterone by 3β-HSD, and progesterone is converted into dehydroepiandrosterone by CYP17A1 in the endoplasmic reticulum and finally metabolised to testosterone by 17β-HSD (Aghazadeh et al., 2015; Dolatkhah et al., 2022). Alterations in the expression of key enzymes can directly affect T synthesis. T plays a vital role in maintaining the normal viability of spermatozoa, promoting sperm development and maturation, and facilitating the proper functioning of the reproductive system. Collectively, these factors contribute to the essential foundation of male fertility (Witherspoon and Flannigan, 2022).

The impairment of reproductive function is a notable adverse effect associated with cyclophosphamide (CP), a nonspecific cytotoxic drug commonly used in tumor therapy (Chen et al., 2017). Research suggests that CP can cause substantial damage to the testes and increase the risk of oligospermia or azoospermia, ultimately leading to infertility. Consequently, the creation of an animal model for studying spermatogenic disorders using CP has become a widely adopted approach for exploring the underlying mechanisms and assessing potential treatment interventions. This method offers the advantages of rapid onset, short duration, and enhanced stability (Wang et al., 2019).

In Chinese medicine, *Cynomorium songaricum* Rupr (CS) is primarily used to treat impotence and renal yang deficits (Nickrent et al., 2005; Tao et al., 1999). Contemporary pharmacological studies have identified terpenoids, organic acids, and flavonoids as the active chemical constituents of CS extracts (Wei et al., 2019; Jiang et al., 2001). These constituents exert various pharmacological effects, including fertility enhancement, immune system stimulation, antiviral properties, and nervous system protection (Tian et al., 2019; Xue et al., 2018; Wang et al., 2017). The aqueous extract of CS has been observed to substantially increase sperm count and viability while reducing the number of abnormal spermatozoa (Abd el-Rahman et al., 1999). Furthermore, the CS extract has been shown to directly affect seminiferous tubules, leading to an increase in the wet weight of animal testes and increase testosterone levels (Abd el-Magied et al., 2001). However, there is a lack of published studies on the specific mechanisms through which CS improves reproductive dysfunction, highlighting the need for further research in this area.

**FIGURE 1:**
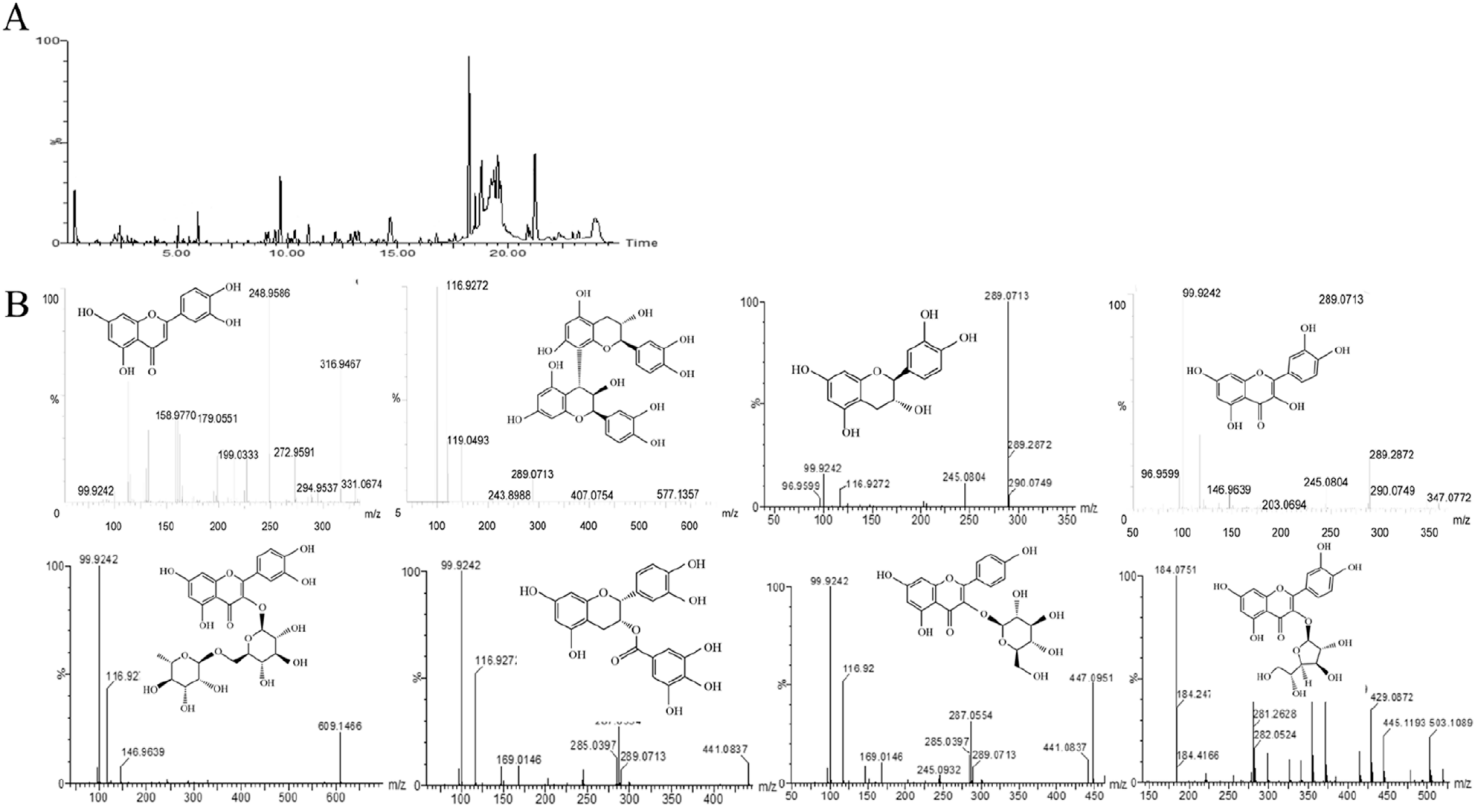
Main components of CSF. **(A)** Total ion flow diagram (negative spectrum) of CSF using LC-MS/MS **(B)** Secondary mass spectra and chemical structure of CSF screened for chemical constituents. CSF, *Cynomorium songaricum* Rupr. flavonoid.

We investigated the protective effects of CS flavonoids (CSF) in male mice with impaired reproductive function. Additionally, we examined the mechanism through which CSF ameliorates reproductive dysfunction and stimulates the proliferation of TM3 cells. The main constituents of the CSF were identified using LC-MS/MS analysis (Figure 1), laying the groundwork for the future development and exploration of CS.

## Materials and methods

### Preparation and separation of CSF

The dried fleshy stems of CS were collected from the Altay region in the Xinjiang Autonomous Region. Professor Zhang Hui from the Changchun University of Traditional Chinese Medicine identified this plant. To extract its components, a feed-to-liquid ratio of 1:30 was used with 50% ethanol refluxed three times at 100°C. The resulting extracts were evaporated, concentrated, and freeze-dried. The extracts were further enriched using an AB-8 macroporous adsorbent resin to obtain CSF (91.3% enriched flavonoid-subfraction).

### LC-MS/MS

Liquid phase conditions:The separation was carried out on an ACQUITY UPLC®BEH C18 column (2.10 mm*50 mm, 1.7 µm). Mobile phase: A: 0.01% formic acid-acetonitrile, B: 0.01% formic acid-water, injection volume 2 µL. Gradient elution conditions Table 1.

**TABLE 1:** Gradient elution conditions.

Time (min)	Flow rate mL/min	Acetonitrile (A) (%)	H_2_O (B) (%)
0	0.4 mL/min	5	95
2	0.4 mL/min	20	80
5	0.4 mL/min	40	60
15	0.4 mL/min	60	40
18	0.4 mL/min	90	10
23	0.4 mL/min	95	5
25	0.4 mL/min	5	95

Mass spectrometry conditions: Through the ESI ion source, using positive, negative ions, sensitivity analysis mode (ESI+, ESI-), MSEcontinuum mode scanning, scanning time of 0.5 s, low-energy ionisation collision energy of 6 V; high-energy collision energy of 10–30 V, cone hole voltage of 30 V; scanning range 40–1500 Da.

Sample treatment: Flavonoid extracts were dissolved in methanol by mass spectrometry, filtered through a 0.22 µm filter membrane and injected into the sample.

### Animals

Six groups, each consisting of ten Kunming mice, were randomly selected to participate in the experiment. All animal-related techniques and procedures were authorized by the Jilin Agricultural University Animal Research Committee (ethical code: 20210915001).

1. Group 1 (BG): This group served as the blank control group. Gastric gavage of equal volume of saline daily for 28 days.
2. Group 2 (MG): Mice in this group were assigned to the cyclophosphamide model group. They received intraperitoneal injections of cyclophosphamide (60 mg/kg) for five consecutive days.
3. Group 3 (FL): In this group, the mice were modeled as the total flavonoid low-dose group. They were administered cyclophosphamide as described for the MG group, dissolve flavonoids (45 mg/kg) in physiological saline and administer gastric gavage continuously for 28 days.
4. Group 4 (FM): Mice in this group were assigned to the total medium-dose group. They were administered cyclophosphamide as described for the MG group, dissolve flavonoids (90 mg/kg) in physiological saline and administer gastric gavage continuously for 28 days.
5. Group 5 (FH): Mice in this group were assigned to the total flavonoids high-dose group. They were administered cyclophosphamide as described for the MG group, dissolve flavonoids (180 mg/kg) in physiological saline and administer gastric gavage continuously for 28 days.
6. Group 6 (PG): Mice in this group were assigned to the clomiphene citrate-positive group. They were administered cyclophosphamide according to the MG group, clomiphene citrate (7.5 mg/kg) in physiological saline and administer gastric gavage continuously for 28 days (Pavin et al., 2018; Watcho et al., 2019).

After the last oral administration, mice were euthanized by intraperitoneal injection of sodium pentobarbital. The testicular and epididymal tissues were weighed for subsequent analysis, and blood samples were collected. One testis was used for histological examination, whereas the other was used for RT-PCR and WB assays.

### Calculation of organ indices and sperm density

The testicular index (TI) was calculated using Equation 1: (Predes Fde et al., 2016)

$$\text{TI}=\left(\text{wet weight of testis}/\text{body weight of mice}\right)\times 100\%$$ (1)

Similarly, The epididymal index (EI) was calculated using Equation 2: (Takagi-Morishita et al., 2022)

$$\text{EI}=\left(\text{wet weight of epididymis}/\text{body weight of mice}\right)\times 100$$ (2)

The tail of the left epididymis of mice was taken, rinsed with preheated saline at 37°C, placed in saline at 37°C, and sufficiently cut with scissors, and incubated for 10 min in a 37°C water bath to allow sperm to fully swim out, 10 μL of sperm suspension was added to the sperm counting plate, and the concentration of spermatozoa was observed and recorded under a microscope.

### Hematoxylin-eosin (HE) assay

HE staining was performed as previously described (He et al., 2021). Fresh testicular and epididymal tissues were fixed in 4% paraformaldehyde. After the tissues were appropriately labeled and processed through gradient ethanol dehydration, xylene clearing, paraffin embedding, sectioning, and staining, images of the stained samples were examined and recorded.

### Serum hormone measurement

The experimental animals were subjected to blood collection from the eyeballs, serum was separated by centrifugation at 3,000 rpm for 3 min and stored at −80 °C. Serum hormone levels were determined using enzyme-linked immunoassay kits (Shanghai Preferred Biotechnology) T, LH, GnRH, FSH, and E2 (Wang et al., 2017).

### Semen plasma assay

Blood was collected from the eyeballs after the last dose, and semen was dissected from the seminal vesicle glands, and seminal plasma obtained by centrifugation after liquefaction (Huang et al., 2023; Kerns et al., 2018). Kits (Nanjing Jiancheng Bioengineering Research Institute) were used to measure fructose and ionic zinc levels in seminal plasma.

### Cell culture

Mouse testicular mesenchymal stromal cells (TM3) were provided by the Wuhan Punosai Life Science and Technology Co. Cell culture conditions: 89% Ham’s F12 nutrient medium +10% FBS +1% P/S, 37°C 5% CO_2_.

### Cell viability

The growth of TM3 cells was measured using the CCK-8 assay. TM3 cells were exposed to different concentrations of CSF (12.5, 25, and 50 μg/mL), while 200 μmoL/L BPA served as the MG (Zhang et al., 2022).

### Cellular secretion of T and LH levels

TM3 cells were co-cultured with 200 μmoL/L BPA for 4 h. The drug group was then cultured for 24 h with CSF concentrations of 12.5, 25, and 50 μg/mL. Cells and cell supernatants were separated, and the levels of LH, intracellular T and secreted T in the supernatants were determined by an enzyme immunoassay kit (Shanghai Preferred Biotechnology).

### RT-PCR analysis

Total RNA was isolated from TM3 cells exposed to 200 μmoL/L BPA, TM3 cells treated with 12.5, 25 and 50 μg/mL CSF, and mouse testis tissue using the Ultrapure RNA Extraction Kit (TaKaRa). RT-PCR was performed to assess the relative expression of each gene. The primer information is shown in Table 2.

**TABLE 2:** Primer information.

Primers	Sequence (5ʹ to 3ʹ)	Length (bp)
GAPDH-F	AAC​TTT​GGC​ATT​GTG​GAA​GG	20
GAPDH-R	ACA​CAT​TGG​GGG​TAG​GAA​CA	20
StAR-F	CTG​CTA​GAC​CAG​CCC​ATG​GAC	21
StAR-R	TGA​TTT​CCT​TGA​CAT​TTG​GGT​TCC	24
CYP11A1-F	CGA​TGA​CCT​ATT​CCG​CTT​TGC	21
CYP11A1-R	TGT​GGA​ACA​TCT​GGT​AGA​CGG​C	22
CYP17A1-F	TGG​GCA​CTG​CAT​CAC​GAT​AA	20
CYP17A1-R	GCT​CCG​AAG​GGC​AAA​TAA​CT	20
3β-HSD-F	AGA​AGT​GAC​AGG​CCC​AAA​CT	20
3β-HSD-R	ACA​TGG​ATC​TCA​GGG​CAC​AA	20

### WB analysis

Proteins were extracted and their concentrations were measured from TM3 cells exposed to 200 μmol/L BPA, TM3 cells treated with 12.5, 25, and 50 μg/mL CSF, and mouse testicular tissue using a total protein extraction kit and a protein concentration determination kit (Bestbio). Protein blot analysis was conducted, and image processing was performed using PictureJ software.

### Statistical analyses

Statistical analyses were performed using the SPSS software (version 17.0). The results are represented as mean ± SD. Group comparisons were made using ANOVA, and a *p*-value of less than 0.05 was considered statistically significant.

## Results

The main components of CSF were determined by LC-MS/MS.

The primary chemicals present in CS were identified using an LC-MS/MS system, consistent with previous reports (Zhang et al., 2012). Table 3 shows the primary constituents of CSF determined using LC-MS/MS. The negative spectrum of the total ion flow diagram is represented in Figures 1A, B shows the structural equations and secondary mass spectra.

**TABLE 3:** Chemical composition of CSF.

No	Component name	Neutral mass (Da)	Observed (m/z)	Observed RT (min)	Adducts	Formula
1	Luteolin	286.04774	331.0674	0.36	+HCOO^−^, -H	C_15_H_10_O_6_
2	Proanthocyanidins	578.14243	577.1357	2.15	−H	C_30_H_26_O_13_
3; 4	(−)-Catechin/(+)-Catechin hydrate	290.07904	289.0713	2.86/2.4	−H	C_15_H_14_O_6_
5	Quercetin	302.04265	347.0772	2.88	−H, +HCOO^−^	C_15_H_10_O_7_
6	Rutin	610.15338	609.1466	3.47	−H	C_27_H_30_O_16_
7	(−)-Epicatechin gallate	442.09	441.0837	3.62	−H	C_22_H_18_O_10_
8	Astragalin	448.10056	447.0942	3.63	−H	C_21_H_20_O_11_
9	Isoquercitrin	464.09548	503.1089	22.39	+K	C_21_H_20_O_12_

CSF, *Cynomorium songaricum* Rupr. flavonoid.

### Effects of CSF on body mass, T, epididymal index, and sperm count in mice with reproductive impairment

**FIGURE 2:**
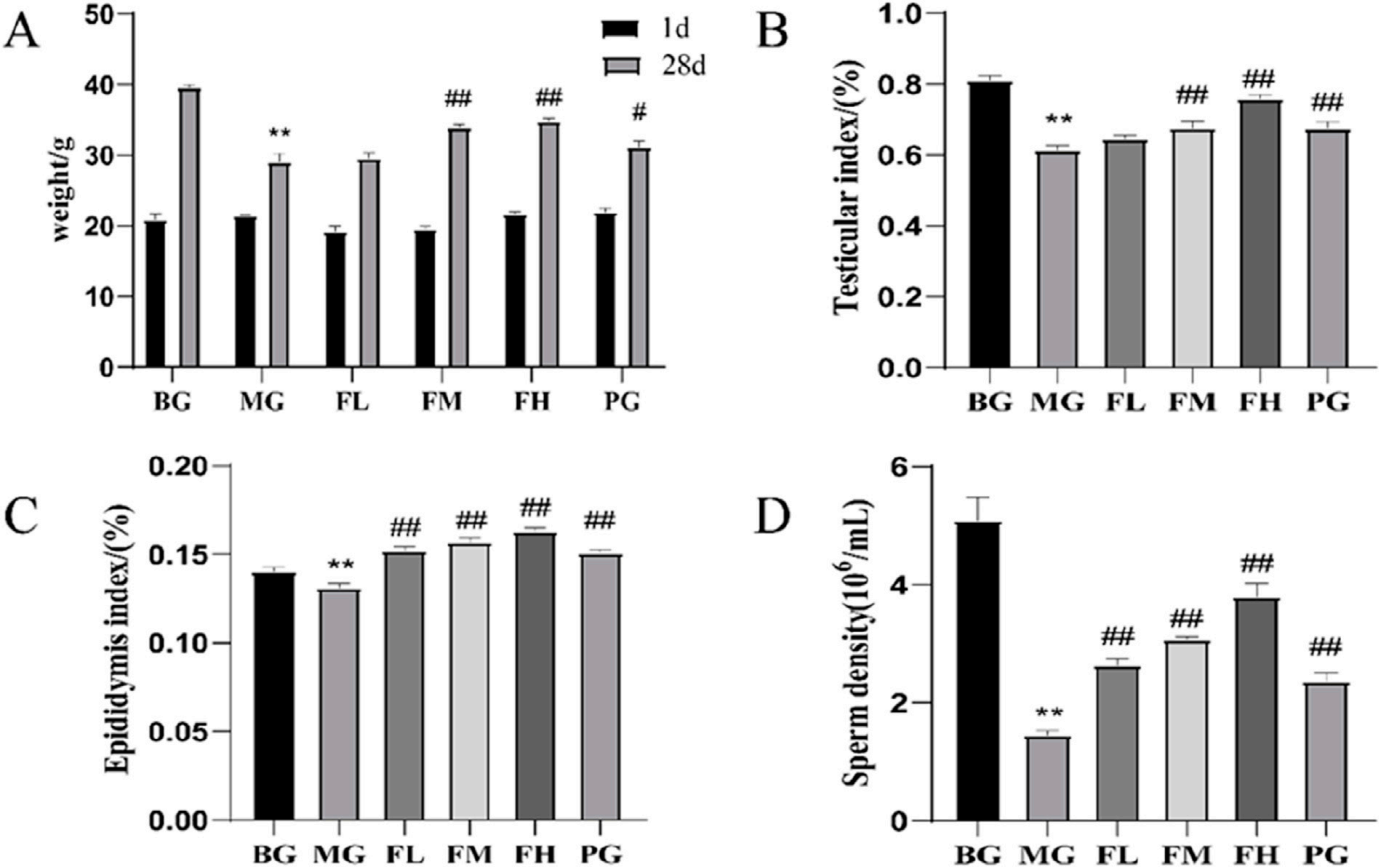
CSF improves **(A)** body mass, **(B)** testicular index, **(C)** epididymal index, and **(D)** sperm count in mice with cyclophosphamide-induced reproductive impairment. **P < 0.01 vs. BG; #*P* < 0.05, ##*P* < 0.01 vs. MG, n = 10. BG, blank group; CSF, *Cynomorium songaricum* Rupr. flavonoid; FL, low-dose flavonoid group; FM, medium-dose flavonoid group; FH, high-dose flavonoid group; MG, model group; PG, clomiphene citrate-positive group.

After 28 days of modeling and therapy, the MG group exhibited significantly lower body mass, testicular and epididymal indices, and sperm count than those of the BG group. Compared with the MG group, the body masses of mice in the FM and FH groups were significantly higher (Figure 2A). All the other dosing groups displayed increased testicular and epididymal indices, as depicted in Figures 2B, C. Furthermore, all other dosing groups exhibited significantly higher sperm counts than that in the MG group (Figure 2D).

### CSF regulates serum hormone levels and seminal plasma energy substance content

**FIGURE 3:**
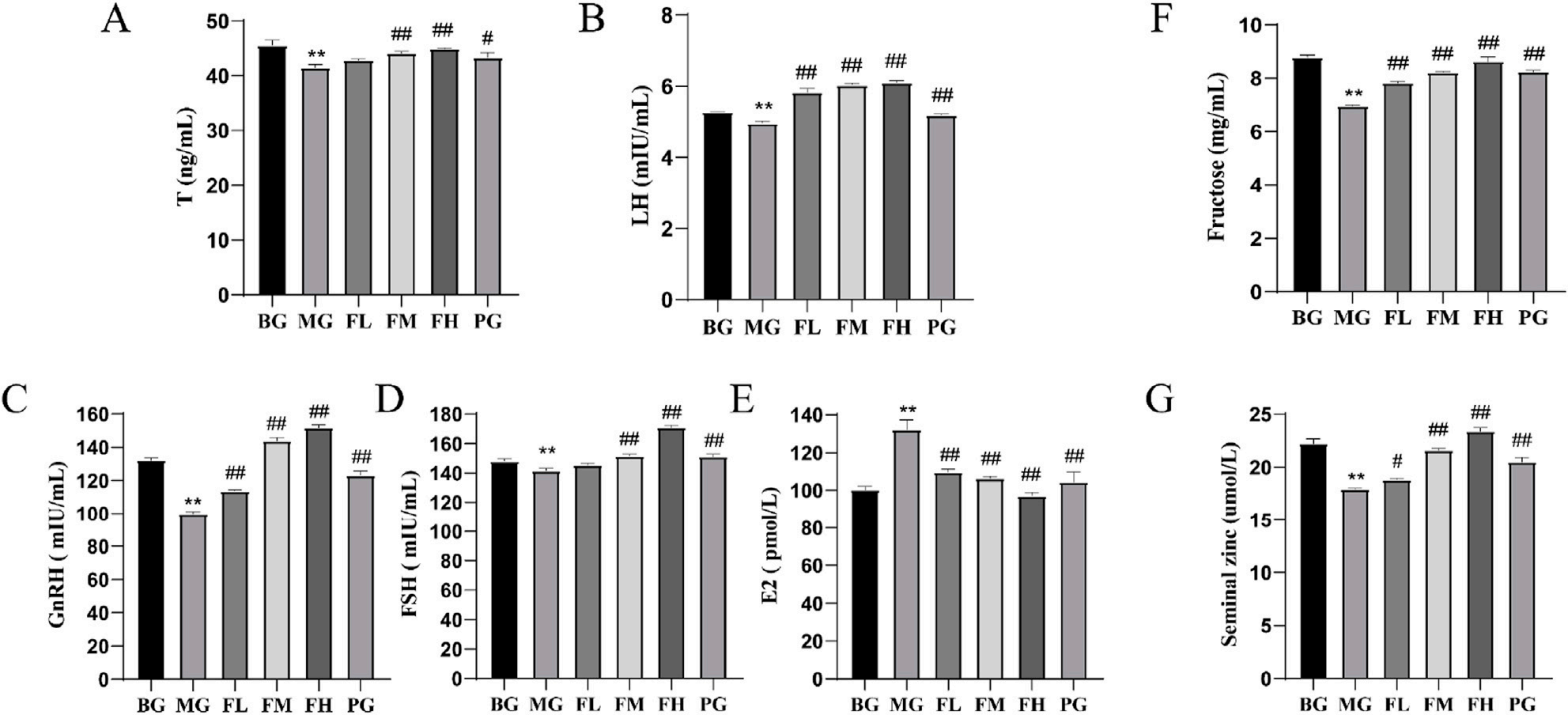
Effects of CSF on sex hormone levels and seminal plasma energetics in mice. **(A)** T, **(B)** LH, **(C)** GnRH, **(D)** FSH, **(E)** E2, **(F)** fructose, and **(G)** zinc ions. **P < 0.01 vs. BG; #*P* < 0.05, #*P* < 0.05 vs. 0.05; ##*P* < 0.01 vs. MG, n = 3. BG, blank group; CSF, *Cynomorium songaricum* Rupr. flavonoid; FL, low-dose flavonoid group; FM, medium-dose flavonoid group; FH, high-dose flavonoid group; LH, luteinizing hormone; MG, model group; PG, clomiphene citrate-positive group; T, testosterone.

The ability to regulate hormone levels along the HPG axis. Compared with the BG group, the MG group showed significantly lower serum levels of T, LH, GnRH, and FSH (*P* < 0.01) (Figures 3A–D). Conversely, serum E2 levels were significantly higher in the MG group than in the BG group but were significantly lower in all other dosing groups compared with those in the MG group (Figure 3E). Furthermore, seminal fructose and zinc levels were significantly higher (*P* < 0.05; *P* < 0.01) (Figures 3F, G) in all the other dosing groups than those in the MG group. These findings indicate that CSF can enhance the quality of mouse spermatozoa by elevating the concentrations of fructose and zinc in seminal plasma.

### CSF improves testicular and epididymal histopathology

**FIGURE 4:**
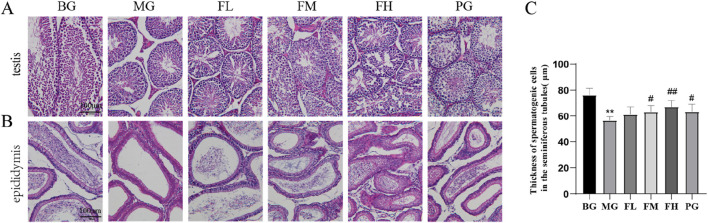
Effect of CSF on testicular and epididymal tissues. Sections of **(A)** testis and **(B)** epididymis from each group of mice were analyzed using HE staining. **(C)** Measurement of spermatogenic cell layer thickness. **P < 0.01 vs. BG; #*P* < 0.05, ##*P* < 0.01 vs. MG, n = 10. BG, blank group; CSF, *Cynomorium songaricum* Rupr. flavonoid; FL, low-dose flavonoid group; FM, medium-dose flavonoid group; FH, high-dose flavonoid group; MG, model group; PG, clomiphene citrate-positive group.

In the BG group, the testes exhibited a well-covered plasma membrane, a high number of seminiferous tubules, and an interstitium consisting of loose connective tissue between the tubules. However, in the MG testes, a lower number of spermatogonia, spermatocytes, and spermatozoa was observed compared with those in the BG group, indicating an impaired maturation process of spermatogonia. Notably, the thickness of the spermatogonial cell layer in the MG group was significantly decreased (*P* < 0.001) (Figure 4C) (Kerns et al., 2018). A few seminiferous tubules exhibit a small number of disorganized spermatocytes, and in other tubules, no spermatozoa were observed, effectively reproducing animal models (Kerns et al., 2018). Overall, spermatogenesis was significantly reduced in the testes.

In the groups administered with total flavonoids, the content of spermatogonial cells in the testis and the distribution of spermatozoa showed varying degrees of improvement among the different dose groups. The FM and FH groups were more effective in enhancing the spermatogenic capacity of the testicular spermatogonia. Furthermore, the thickness of spermatogonial cells in the testes improved in the PG group, although the degree of improvement was not as pronounced as that in the FH group (Figure 4A).

A well-arranged distribution of epididymal ducts containing numerous spermatozoa was observed in the epididymis of the BG group. However, the MG group displayed a significant decrease in the number of spermatozoa distributed in the lumen of the epididymal ducts compared with that in the BG group. In contrast, the total flavonoids in the groups exhibited a significant improvement in the number of spermatozoa distributed in the epididymal ducts compared with that in the MG group (Figure 4B).

### Assessment of steroidogenic enzyme-related genes and protein expression in testicular tissues

**FIGURE 5:**
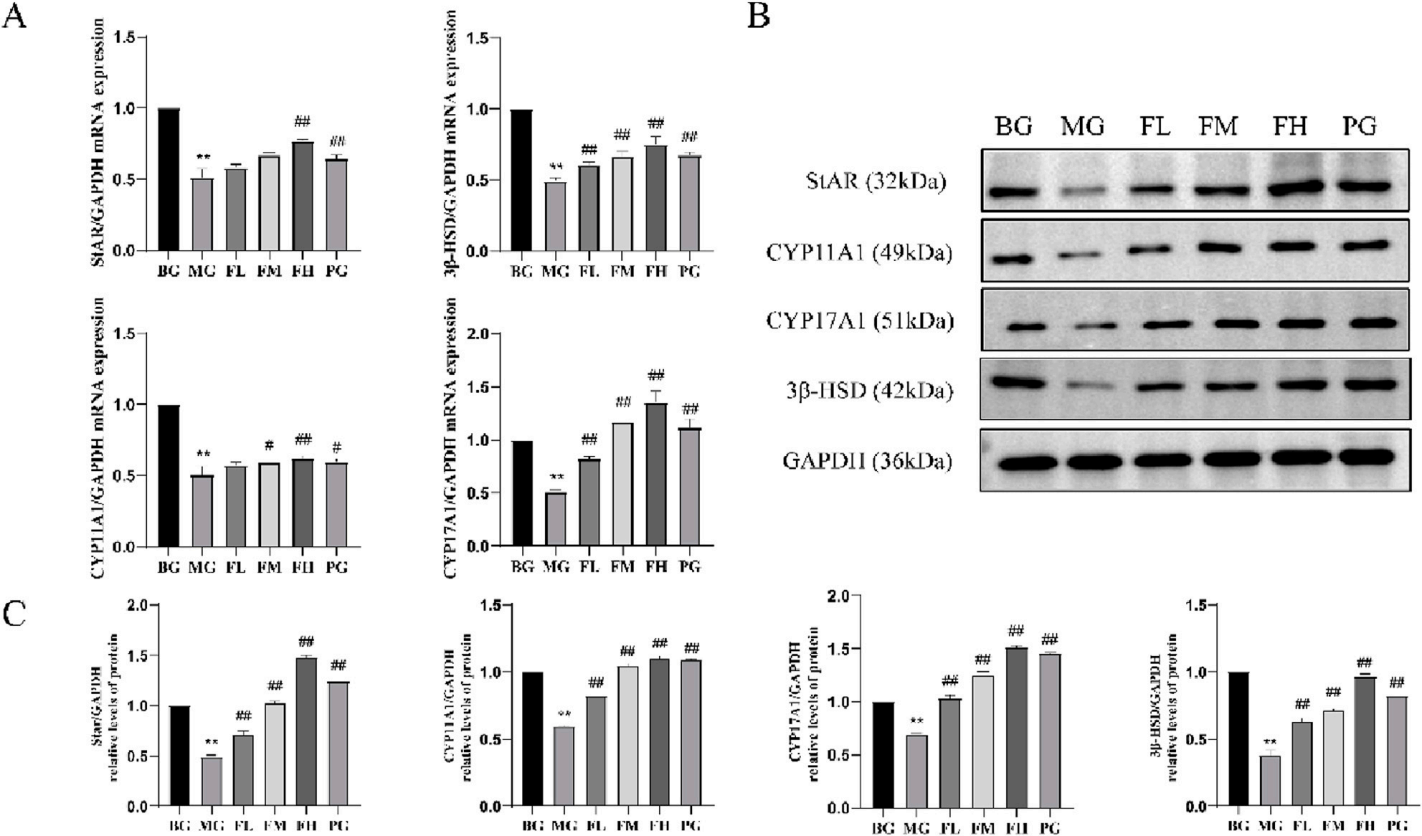
Effect of CSF on testicular tissue steroidogenic enzyme-related gene and protein expression. **(A)** CSF promotes testicular tissue steroidogenic enzyme mRNA expression, **(B,C)** CSF promotes testicular tissue steroidogenic enzyme protein expression. *P < 0.05, **P < 0.01 vs. control; #*P* < 0.05, ##*P* < 0.01 vs. model. BG, blank group; CSF, *Cynomorium songaricum* Rupr. flavonoid; FL, low-dose flavonoid group; FM, medium-dose flavonoid group; FH, high-dose flavonoid group; MG, model group; PG, clomiphene citrate-positive group.

CSF administration resulted in the upregulation of steroidogenic enzyme-related genes and proteins, including StAR, CYP11A1, CYP17A1, and 3β-HSD, in mouse testicular tissues. Compared with the BG group, the MG group exhibited a significant reduction in the expression of these genes and proteins. However, the FL, FM, FH, and PG groups demonstrated a significant increase in the expression of steroidogenic enzyme-related genes and proteins in their testicular tissues compared with that in the MG group (Figures 5A–C).

### CSF promotes TM3 cell viability and BPA modeling concentrations

**FIGURE 6:**
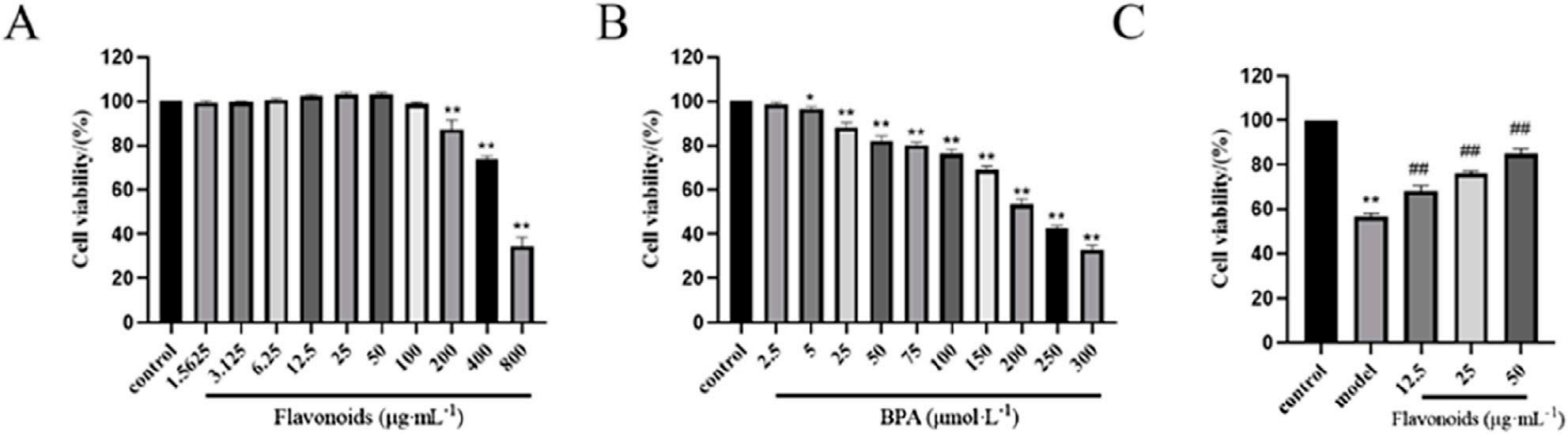
Effects of CSF and BPA on TM3 cell viability *in vitro*. **(A)** CSF promoted the proliferation of TM3 cells (selection of concentrations 12.5, 25, and 50 μg/mL). **(B)** BPA effectively inhibited the proliferation of TM3 cells (selected concentration 200 μmoL/L). **(C)** CSF improved BPA inhibited TM3 cell viability. *P < 0.05, **P < 0.01 vs. control; #*P* < 0.05, ##*P* < 0.01 vs. model, n = 8. CSF, *Cynomorium songaricum* Rupr. flavonoid.

The proliferation of TM3 cells was assessed using CCK-8, revealing that cell viability reached its peak at a concentration of 50 μg/mL CSF. However, cell viability started to decline as the concentration increased (Figure 6A). Additionally, the inhibitory effect of BPA on TM3 cells was evaluated, demonstrating that the cell survival rate was 53.57% ± 2.08% after a 4-h exposure to 200 μmoL/L BPA (Figure 6B). Hence, a 4-h incubation period with 200 μmoL/L BPA was selected for subsequent experiments. Comparatively, in the presence of BPA-induced injury, CSF concentrations of 12.5, 25, and 50 μg/mL demonstrated a significant dose-dependent increase in the viability of TM3 cells when compared with the MG (*P* < 0.05; *P* < 0.01) (Figure 6C). Therefore, CSF concentrations of 12.5, 25, and 50 μg/mL were chosen for further trials.

### CSF alleviates BPA inhibition of T and LH secretion by TM3 cells

**FIGURE 7:**
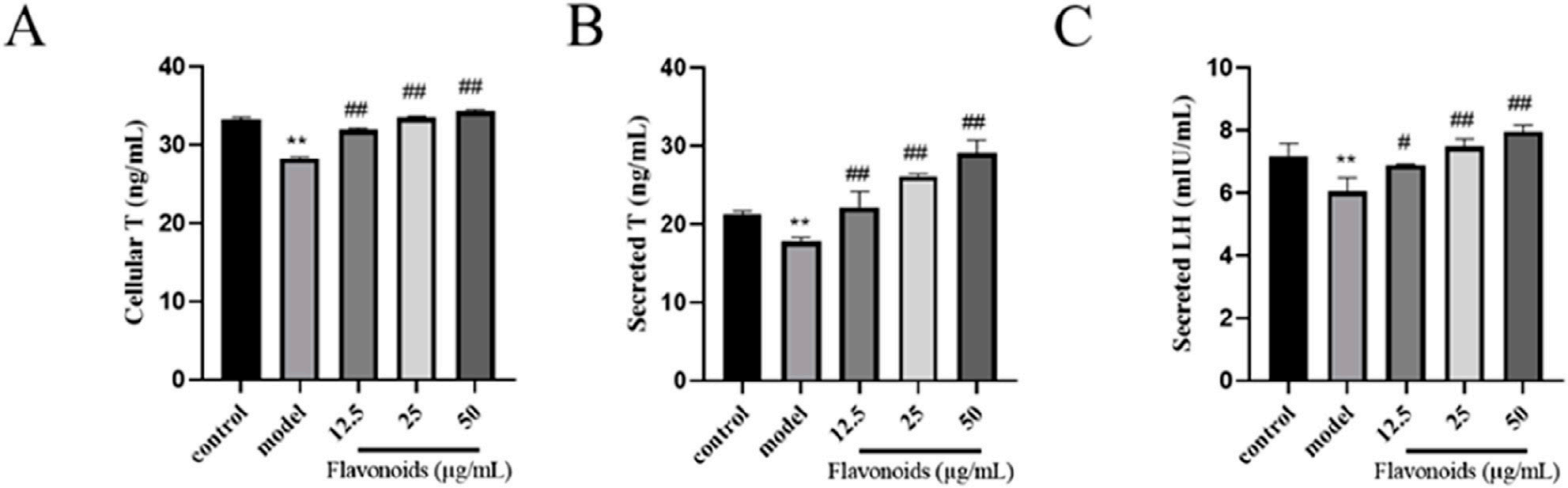
Effect of CSF on T and LH secretion in TM3 cells. ELISA for **(A)** intracellular T levels, **(B)** secreted T levels, and **(C)** secreted LH levels in TM3 cells. **P < 0.01 vs. BG; #*P* < 0.05, ##*P* < 0.01 vs. MG, n = 3. BG, blank group; CSF, *Cynomorium songaricum* Rupr. flavonoid; LH, luteinizing hormone; MG, model group; T, testosterone.

After 4 h of stimulation with 200 μmol/L BPA, the intracellular T level in the MG was significantly lower than that in the BG (*P* < 0.01) (Figure 7A). However, after 24 h of pharmacological protection with varying concentrations of CSF, the intracellular T content showed a dose-dependent increase in each dose group, with significantly higher levels than those in the MG (*P* < 0.01). The higher the CSF concentration within the safe dosage range, the higher the intracellular T content (Figures 7B, C). Luteinizing hormone (LH) is a glycoprotein gonadotropin secreted by adenopituitary cells, which contributes to the synthesis and release of T from TM3 cells. Furthermore, upon stimulation with 200 μmol/L BPA for 4 h, the secretion of T by TM3 cells in the MG was significantly lower than that observed in the BG (*P* < 0.01) (Figures 7B, C). However, in the presence of CSF within a safe dose range, increasing concentrations of the drug corresponded with higher cellular secretion levels of T. These results indicate that CSF exhibits a certain degree of improvement in alleviating the inhibition of TM3 cells secretion of both T and LH by BPA.

### Assessment of steroidogenic enzyme-related genes and protein expression in TM3 cells

**FIGURE 8:**
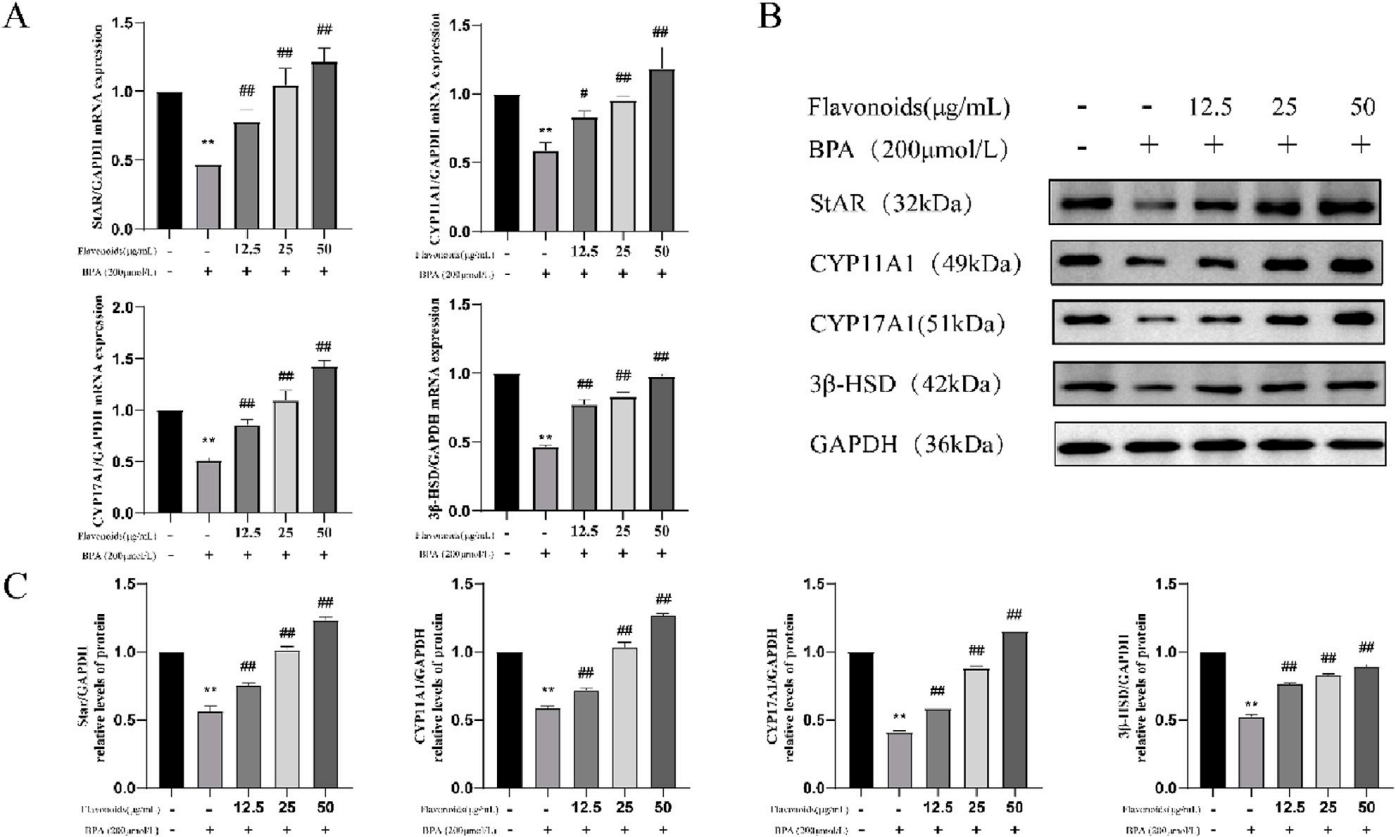
Effect of CSF on the expression of TM3 cell steroidogenic enzyme-related genes and proteins. **(A)** CSF promotes TM3 cell steroidogenic enzyme mRNA expression, **(B,C)** CSF promotes TM3 cell steroidogenic enzyme protein expression. *P < 0.05, **P < 0.01 vs. control; #*P* < 0.05, ##*P* < 0.01 vs. model. CSF, *Cynomorium songaricum* Rupr. flavonoid.

Administration of CSF effectively reversed the BPA-induced decrease in the expression of steroidogenic enzyme genes and proteins (Figure 8). These findings suggested that BPA treatment negatively affected the T production pathway in TM3 cells, leading to decreased steroidogenic enzyme expression. However, administration of CSF counteracted this effect and restored the expression of steroidogenic enzymes.

## Discussion

Studies have indicated that the CS extract can be used to treat spermatorrhea, premature ejaculation, and erectile dysfunction (Gu et al., 2018; Umamaheswari et al., 2022). CS has been reported to elevate serum T levels, facilitate sexual maturation in animals, and enhance male sexual behavior in rats (Cui et al., 2013). Cyclophosphamide, an alkylating agent commonly used in cancer treatment, is known to inflict severe damage to testicular tissues, leading to oligospermia and infertility (Ghobadi et al., 2017). Studies have demonstrated that cyclophosphamide substantially reduces sperm density and viability, increases sperm malformation rate, and disrupts reproductive hormone levels (Namoju et al., 2021). In this study, we used a mouse model of reproductive dysfunction by administering cyclophosphamide to investigate the protective effects of CSF against cyclophosphamide-induced testicular tissue damage.

Objective markers of reproductive function include pathological changes in the testes and epididymis (Wei et al., 2022). Previous studies have shown that cyclophosphamide causes thinning of the spermatogenic epithelium, reduced epithelial layers, disordered arrangements, and decreased sperm populations in the testicular spermatogenic tubules of rats (Zhang et al., 2013). These results were consistent with our observations. Our MG exhibited successful modeling characterized by a reduced content of components at various levels in spermatogenic cells and observed obstacles in the development and spermatogenic cell maturation processes. In the CSF group, the distribution of spermatozoa and the number of spermatogenic cells in the testes demonstrated varying degrees of improvement, thus enhancing the spermatogenic potential of testicular cells.

The hypothalamic-pituitary-gonadal (HPG) axis plays an integral role in the regulation of human and animal reproduction (Xie et al., 2022), and the process of spermatogenesis in males is highly driven by the HPG axis (Khodamoradi et al., 2020). The regulation of the HPG axis is necessary for the maintenance of normal testicular function in males, including testosterone production and male fertility. The hypothalamus secretes GnRH to stimulate the pituitary gland to synthesize LH and FSH, which in turn maintains the production of T and spermatogenesis in the gonads (Corradi et al., 2016). These hormones maintain proper reproductive function and support physiological homeostasis through feedback regulatory mechanisms. In our mouse model, cyclophosphamide-induced reproductive dysfunction led to significantly elevated E2 levels and significantly reduced serum levels of T, LH, GnRH, and FSH. Conversely, the CSF-administered group exhibited significantly lower E2 levels but significantly higher levels of T, LH, GnRH, and FSH. Clomiphene citrate promotes the secretion of male gonadal hormones and increases serum testosterone concentration, thus clomiphene citrate-positive group had significantly higher T levels. These findings suggest that the CSF can maintain sex hormone levels by regulating the gonadal axis.

T, a crucial sex hormone in male animals, is primarily synthesized and secreted by the testicular mesenchymal cells (Lai et al., 2005). Steroidogenic enzymes play a substantial role in this process by affecting T synthesis and, subsequently, reproductive function (Ge et al., 2021). Our study demonstrated that CSF administration upregulated the mRNA and protein expression levels of steroidogenic enzymes both *in vivo* and *in vitro*, suggesting its potential to mitigate reproductive impairment by modulating the T synthesis pathway. Natural products exhibit various activities and therapeutic effects against various diseases. Single-molecule natural products, such as flavonoids, alkaloids, polysaccharides, and terpenoids, have a wide range of biological activities, many of which have been observed to enhance male sterility by improving testicular function and semen quality (Li et al., 2024). Quercetin ameliorated the toxic effects of fenitrothion in rats via the steroidogenic enzyme pathway and the oxidative stress pathway (Saber et al., 2016). Escin can improve sperm quality in male patients with varicocele by reducing oxidative stress in the testicular tissue, alleviating inflammatory reactions, and promoting tension and contraction after venous wall injury, making it a safe and effective drug (Fang et al., 2010). Epigallocatechin-3-O-gallate [EGCG] has excellent antioxidant activity, and studies have shown that it substantially reduces testicular lesions, sperm malformations, and spermatogenic cell apoptosis (Ge et al., 2015). Yu et al. (2010) Significant and dose-dependent antioxidant and anti-fatigue effects of Cynomorium songaricum flavonoids (rutin, catechin and isoquercitrin) on swimming rats were observed during 10 days of swimming exercise. Experimental results suggest that flavonoid supplementation could not only reduce free radical formation and scavenge free radicals, but also enhance endurance exercise performance by reducing muscle fatigue. Additionally, several studies have shown that certain traditional Chinese herbs, such as *Epimedium brevican, Cuscuta australis, Cistanche deserticola*, and Huangqi-Guizhi-Wuwutang, have promising effects on promoting reproduction (Wang et al., 2023; Ozegbe and Omirinde, 2012; Gu et al., 2016; Zhao et al., 2024). Combining these herbs with CS may further enhance their reproductive-promoting properties via synergistic effects.

*Cynomorium songaricum* is a traditional kidney tonifying and yang enhancing medication commonly used to treat erectile dysfunction, premature ejaculation, and nocturnal emissions. Modern pharmacological studies have shown that *C. songaricum* can increase plasma testosterone levels, promote animal sexual maturity, and enhance animal sexual behavior in young male rats (Yan et al., 1991; Wang et al., 2003). Abd el-Magied et al. (2001) found that *C. songaricum* can significantly improve testicular mass and serum testosterone levels in immature rats. These research results are consistent with the findings of this study. Gu et al. (2021) further analyzed the hormone levels in rats and found that *C. songaricum* can significantly increase testosterone levels and regulate FSH and LH secretion levels in oligoasthenozoospermia rats, leading to the normalization of the negative feedback regulation system of sex hormones in oligoasthenozoospermia rats. This is consistent with our research findings. In addition, Yang et al. (2010) studied that *C. songaricum* has a significant effect on sperm parameters and testicular GDNF expression in rats. The results indicate that traditional Chinese medicine extracts can significantly increase the expression of GDNF at the mRNA and protein levels. Cao et al. (2016) found that *C. songaricum* can significantly increase the sperm count, sperm motility, and serum testosterone levels in oligozoospermia and asthenozoospermia rats. The improvement mechanism may be: 1. By inducing GDNF expression in Sertoli cells of the testes, it promotes the proliferation of undifferentiated spermatogonia, thereby promoting the process of spermatogenesis and increasing the number of sperm in the tail of the epididymis; By promoting testosterone secretion and increasing serum testosterone levels, sperm motility can be improved. Therefore, the mechanism of the reproductive promoting effect of *C. songaricum* needs further research.

## Conclusion

Our study showed that CSF ameliorates reproductive impairment by enhancing the expression of pivotal enzymes involved in synthesizing T. The results show that CSF ameliorates cyclophosphamide-induced reproductive impairment and bisphenol A-induced TM3 cell damage in mice by regulating sex hormone levels in the HPG axis and upregulating the expression of steroidogenic enzymes. This study expands our knowledge of the potential mechanisms by which CS protects male fertility, thereby establishing a theoretical foundation for its potential development as a functional food or pharmaceutical drug.

### Study limitations and suggestions

Due to budget and equipment restrictions, we were unable to explore the impact of individual CSF components on enhancing T production and protecting male fertility. Therefore, we recommend investigating this in future studies.

## Data Availability

The original contributions presented in the study are included in the article/Supplementary Material, further inquiries can be directed to the corresponding authors.
