# Supplementary material for: Cynomorium songaricum Rupr. flavonoids improve cyclophosphamide-induced reproductive function damage by regulating the testosterone synthesis pathway
Source: Front Pharmacol. 2024 Aug 22;15:1457780. doi: 10.3389/fphar.2024.1457780 (PMC11374658; doi:10.3389/fphar.2024.1457780)
Supplement: Supplementary file 1 [file Presentation1.PPTX]

## Slide 1
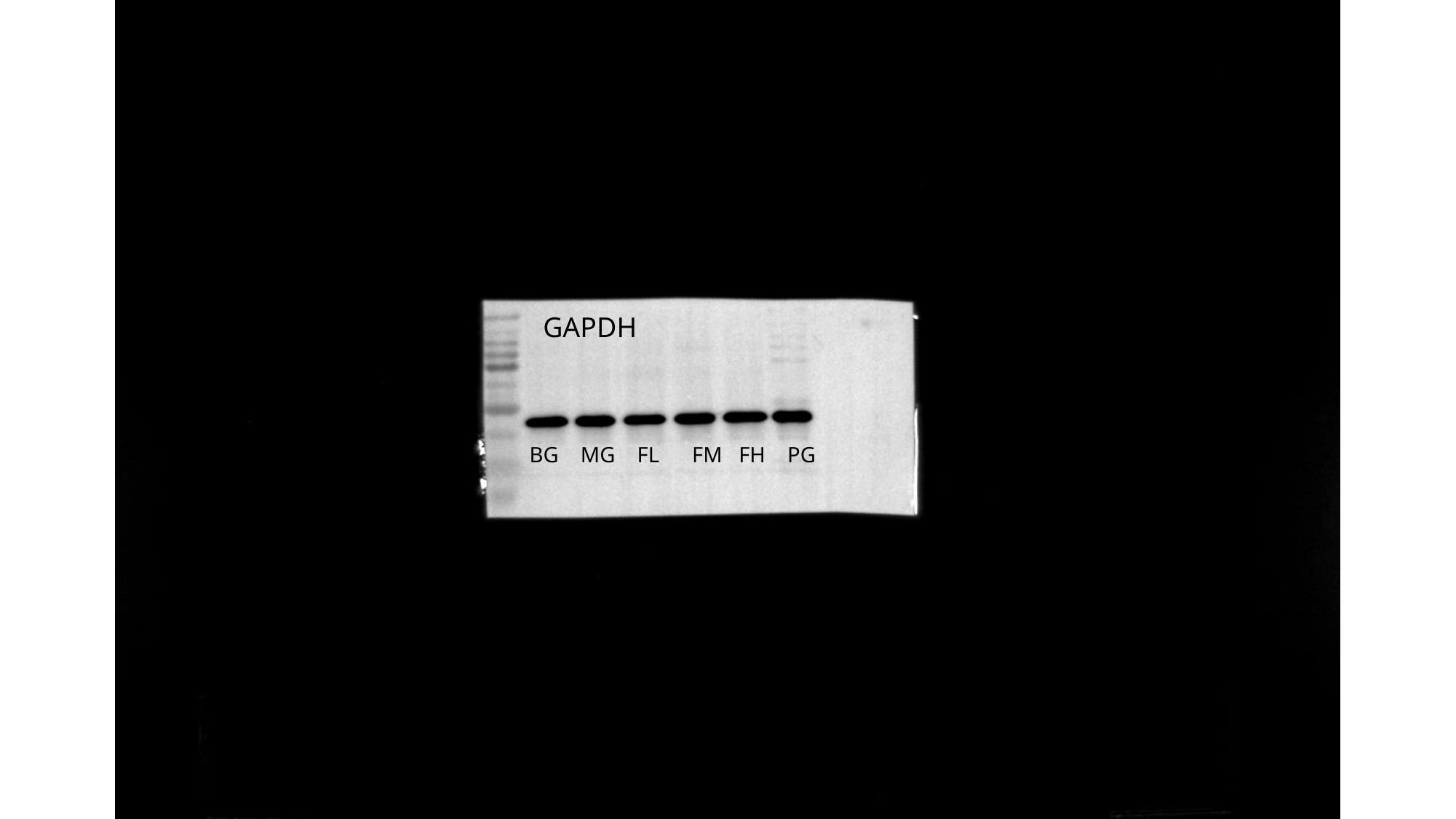

GAPDH
BG MG FL FM FH PG

## Slide 2
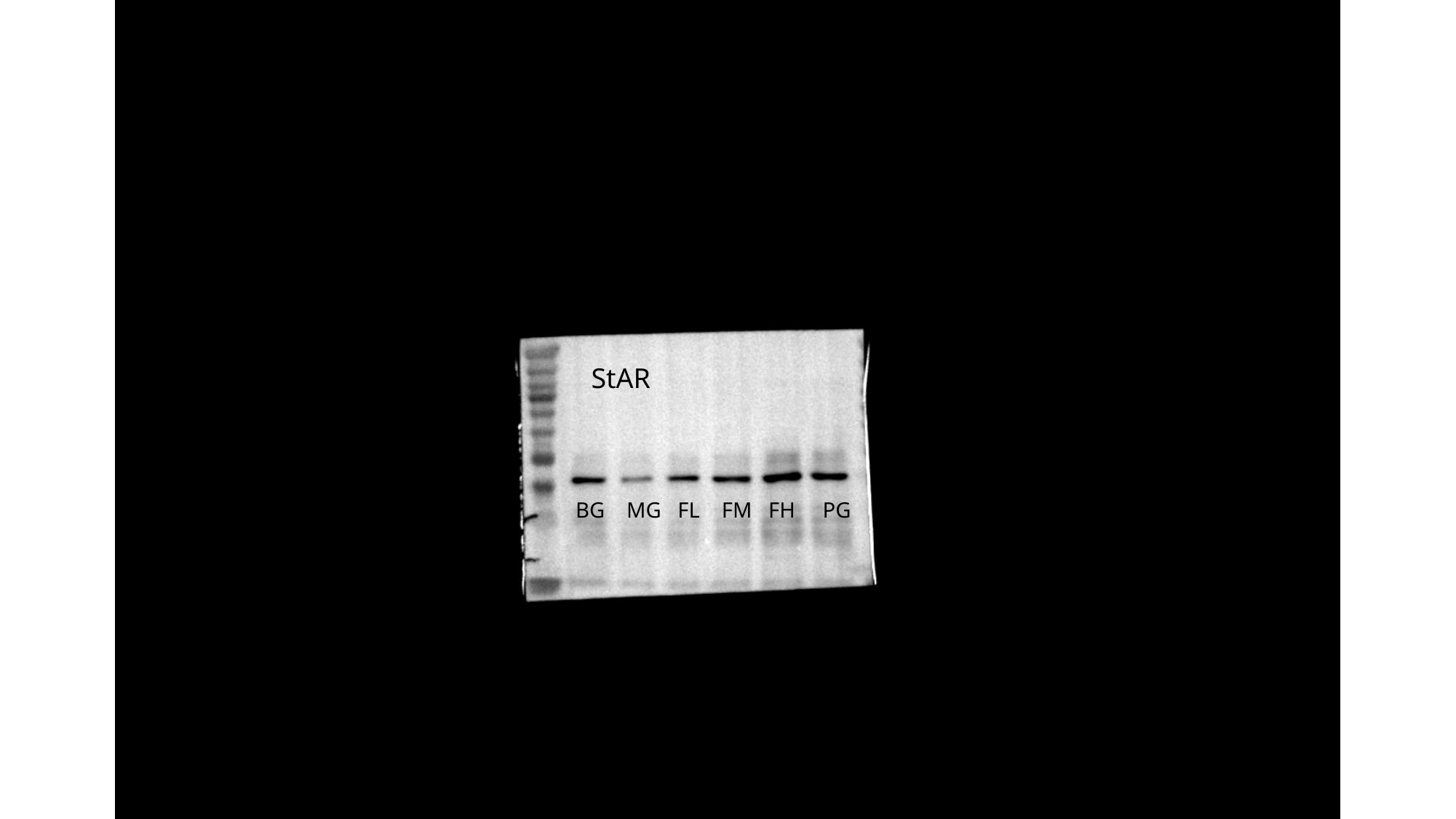

StAR
BG MG FL FM FH PG

## Slide 3
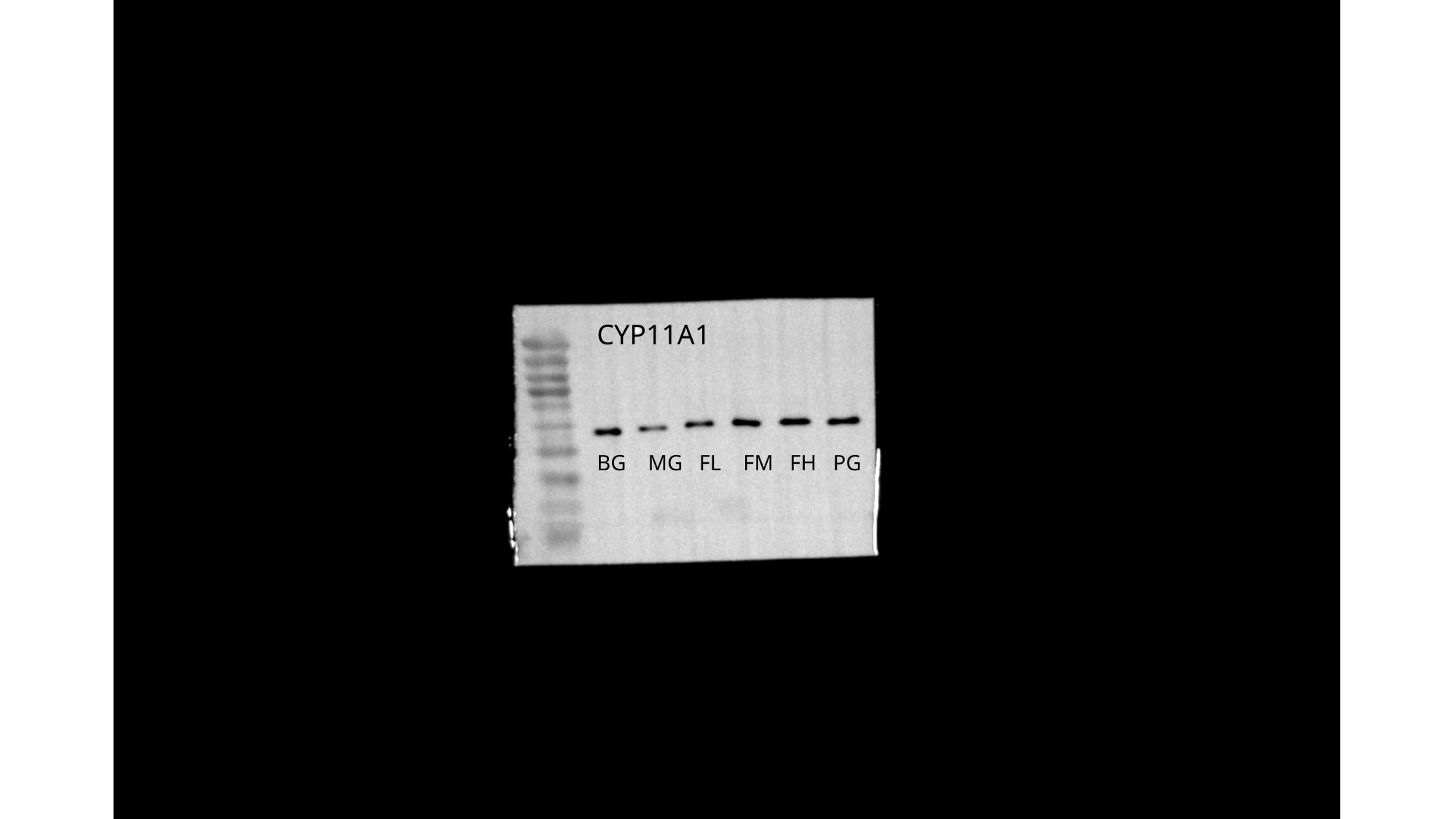

CYP11A1
BG MG FL FM FH PG

## Slide 4
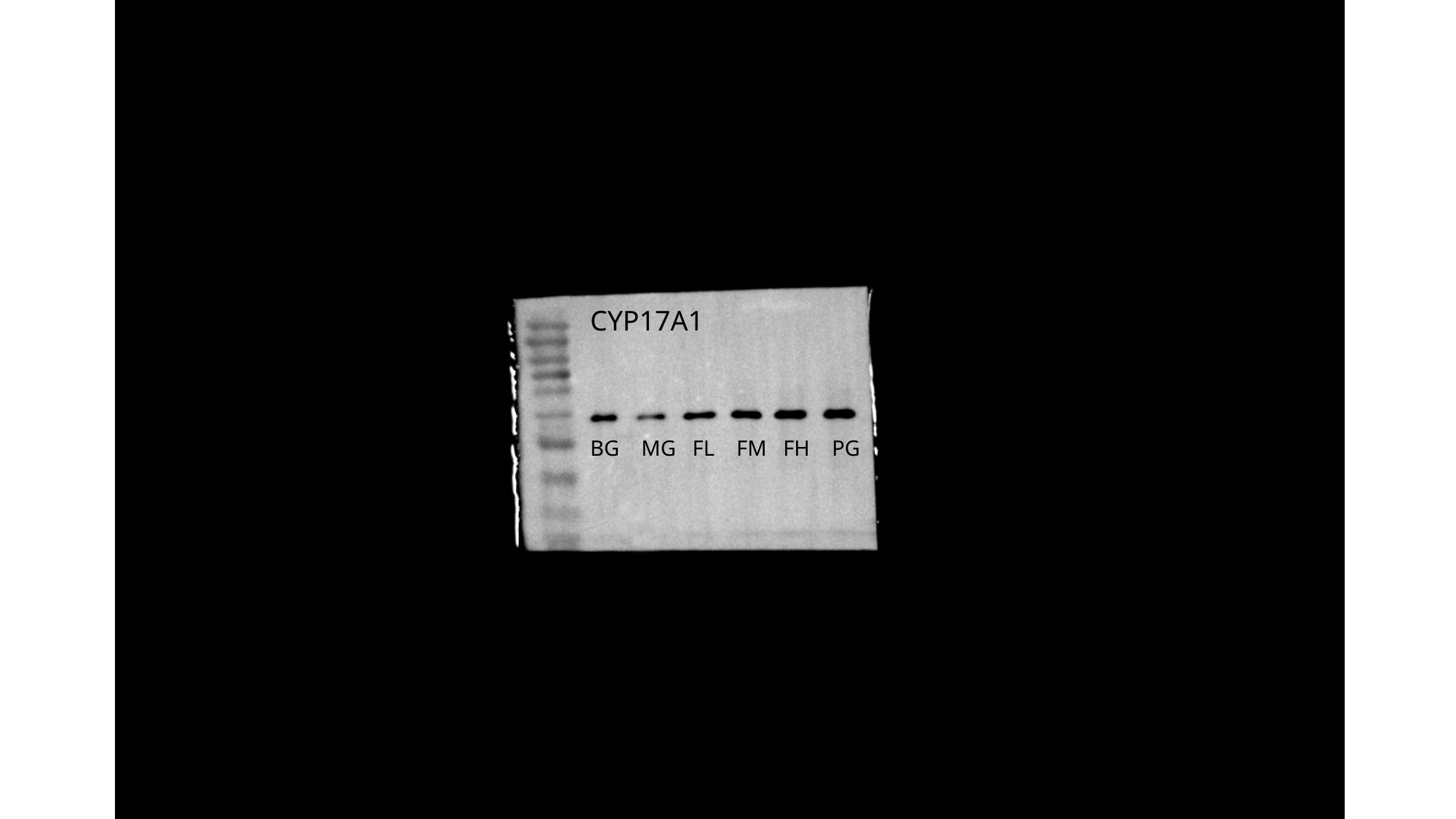

CYP17A1
BG MG FL FM FH PG

## Slide 5
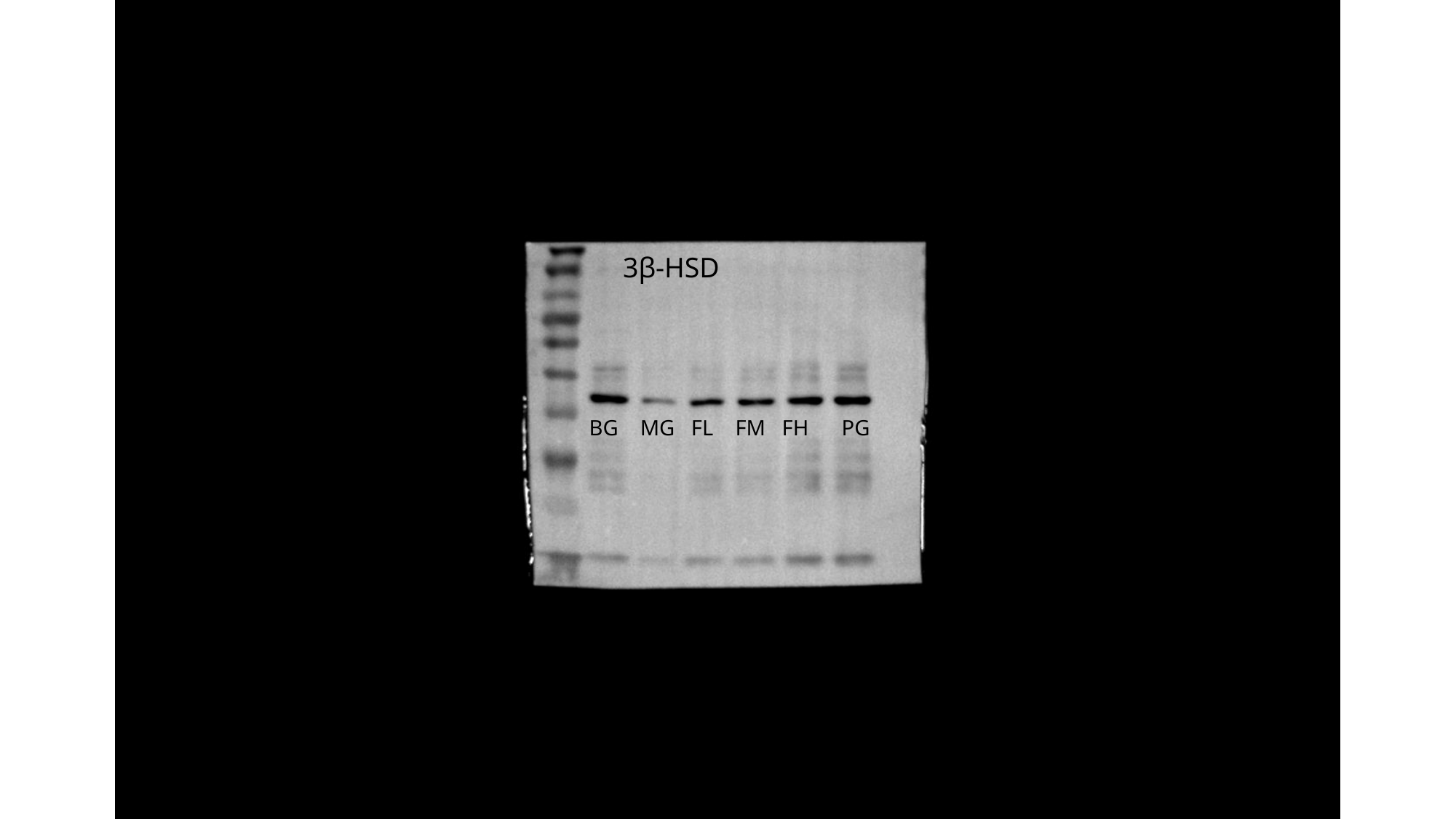

3β-HSD
BG MG FL FM FH PG

## Slide 6
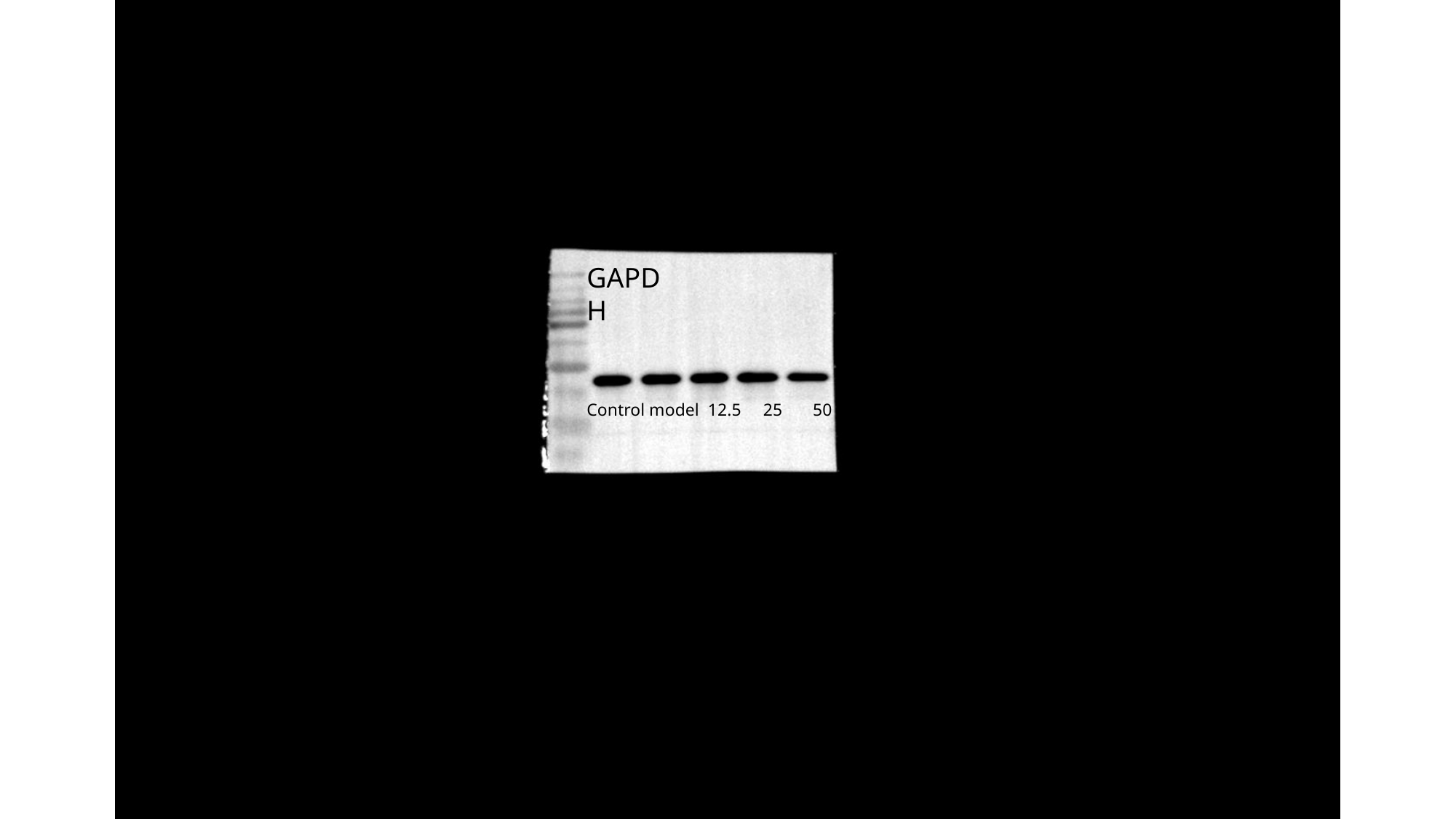

GAPDH
Control model 12.5 25 50

## Slide 7
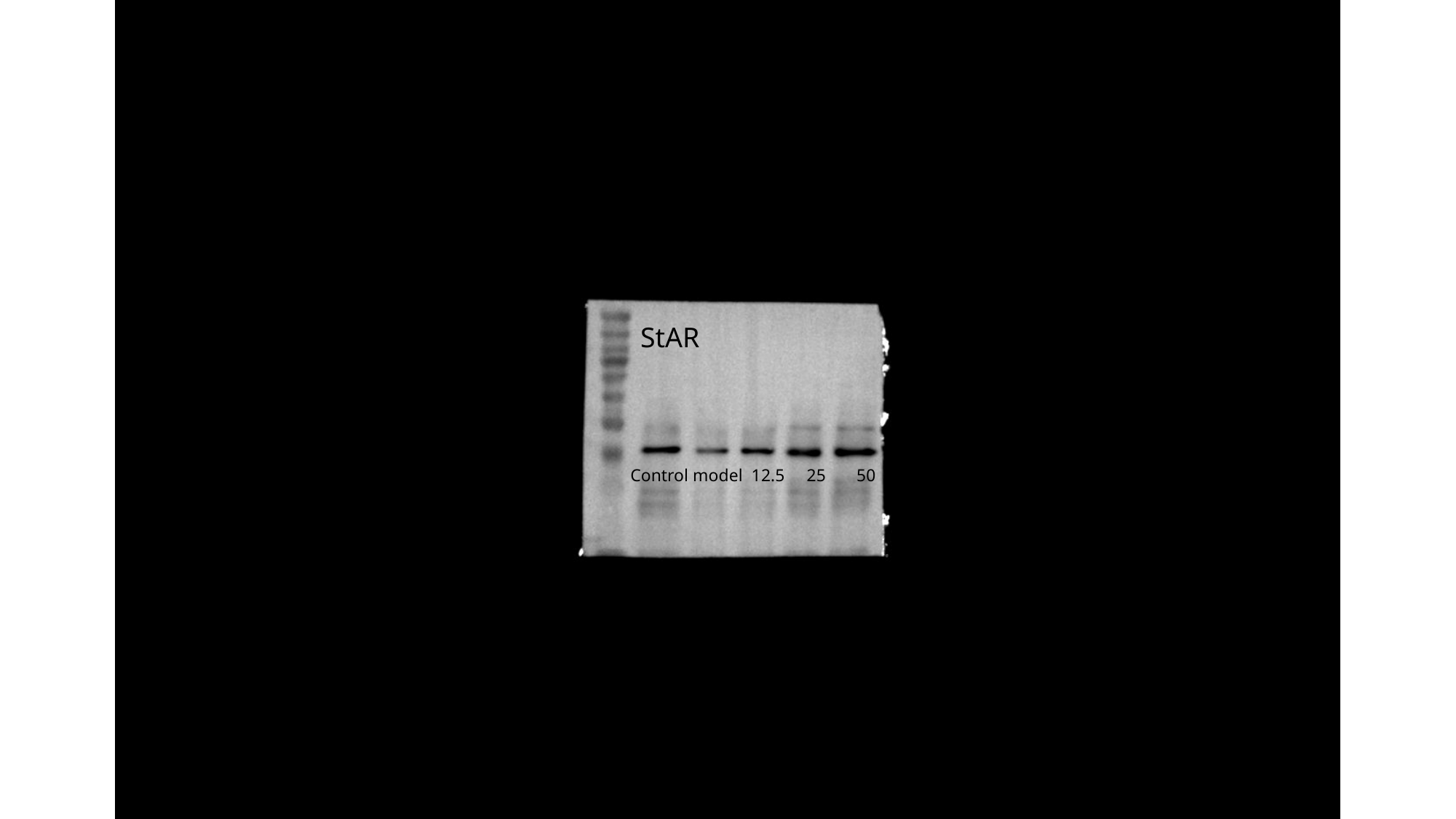

StAR
Control model 12.5 25 50

## Slide 8
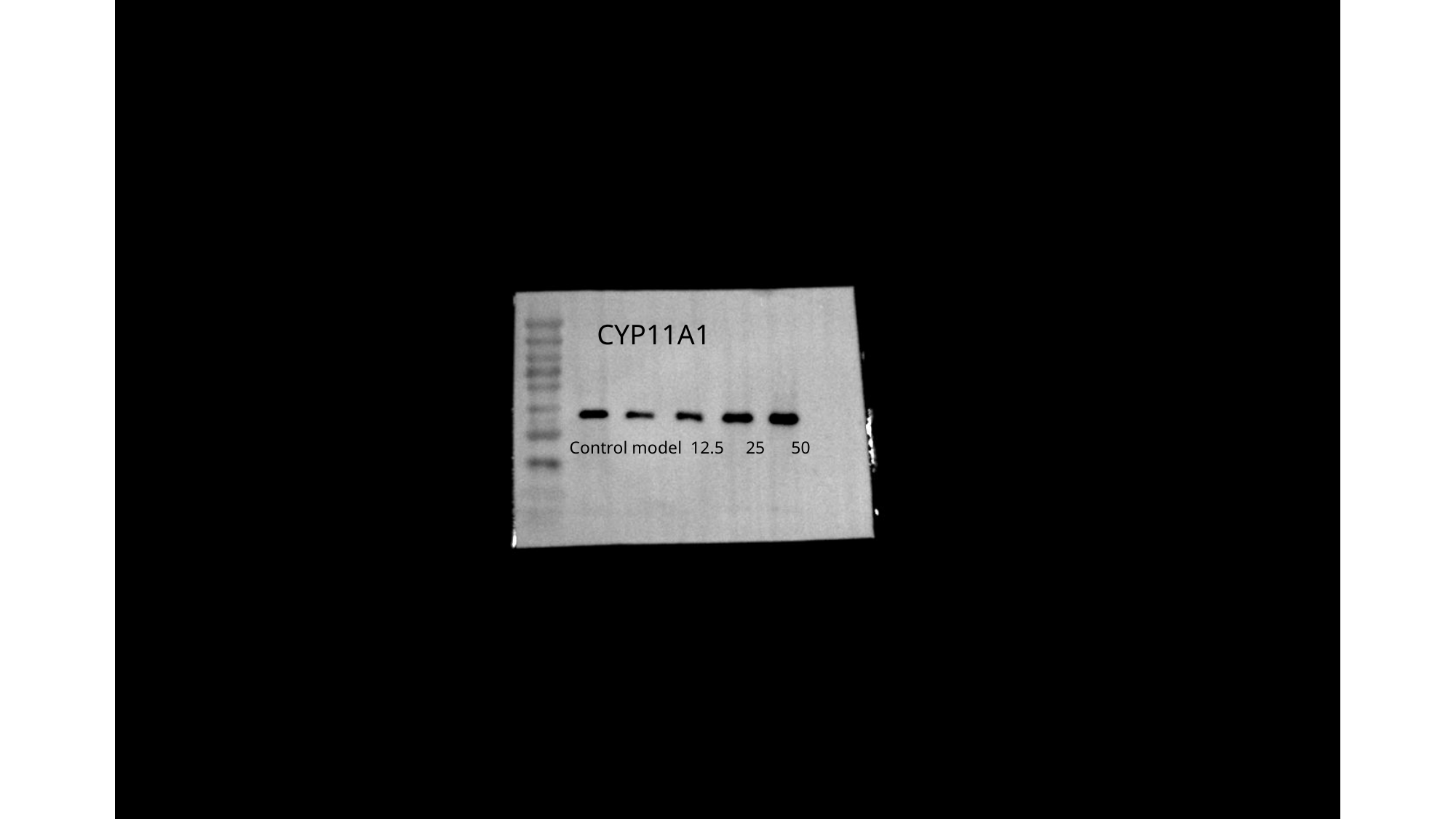

CYP11A1
Control model 12.5 25 50

## Slide 9
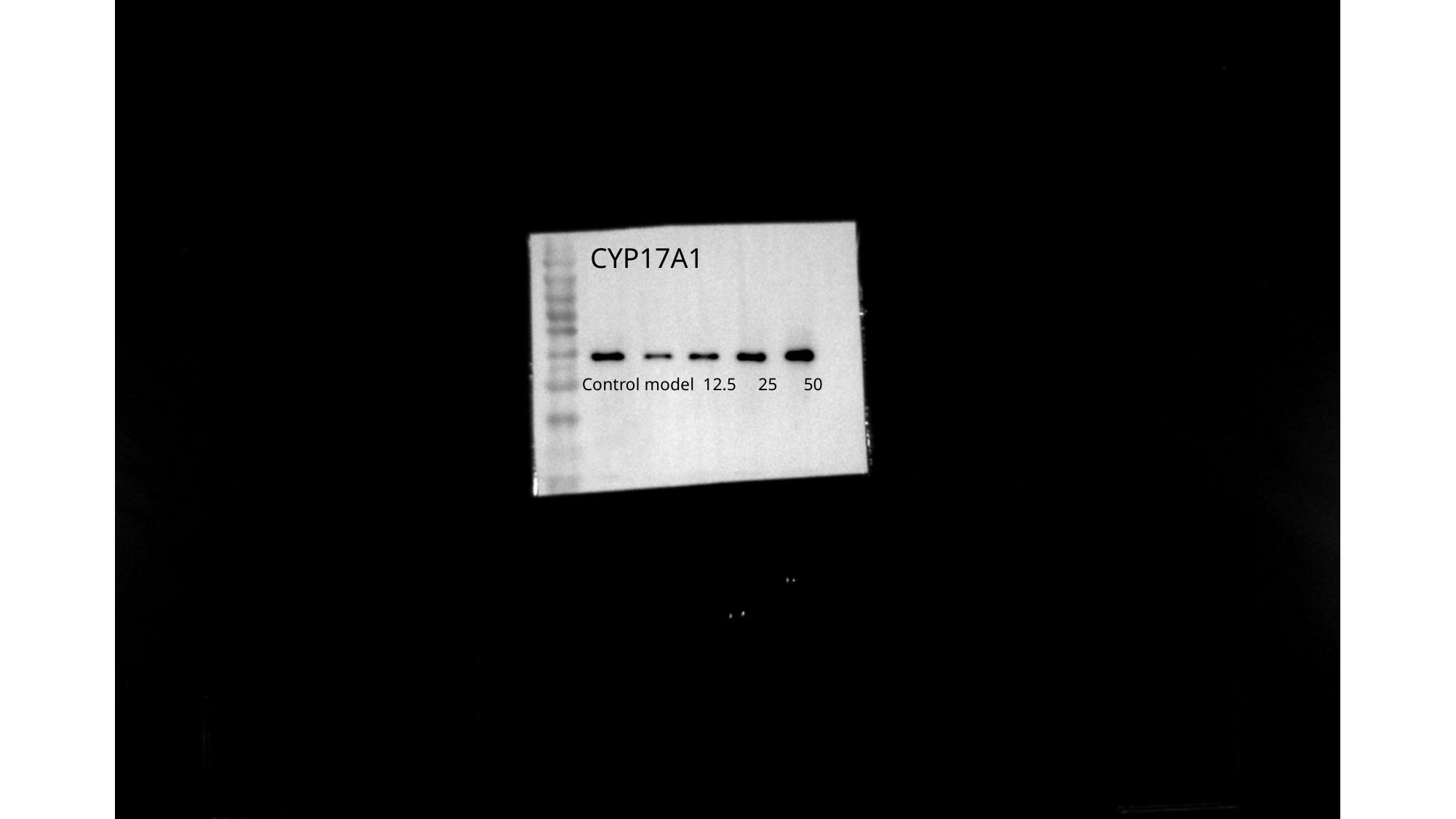

CYP17A1
Control model 12.5 25 50

## Slide 10
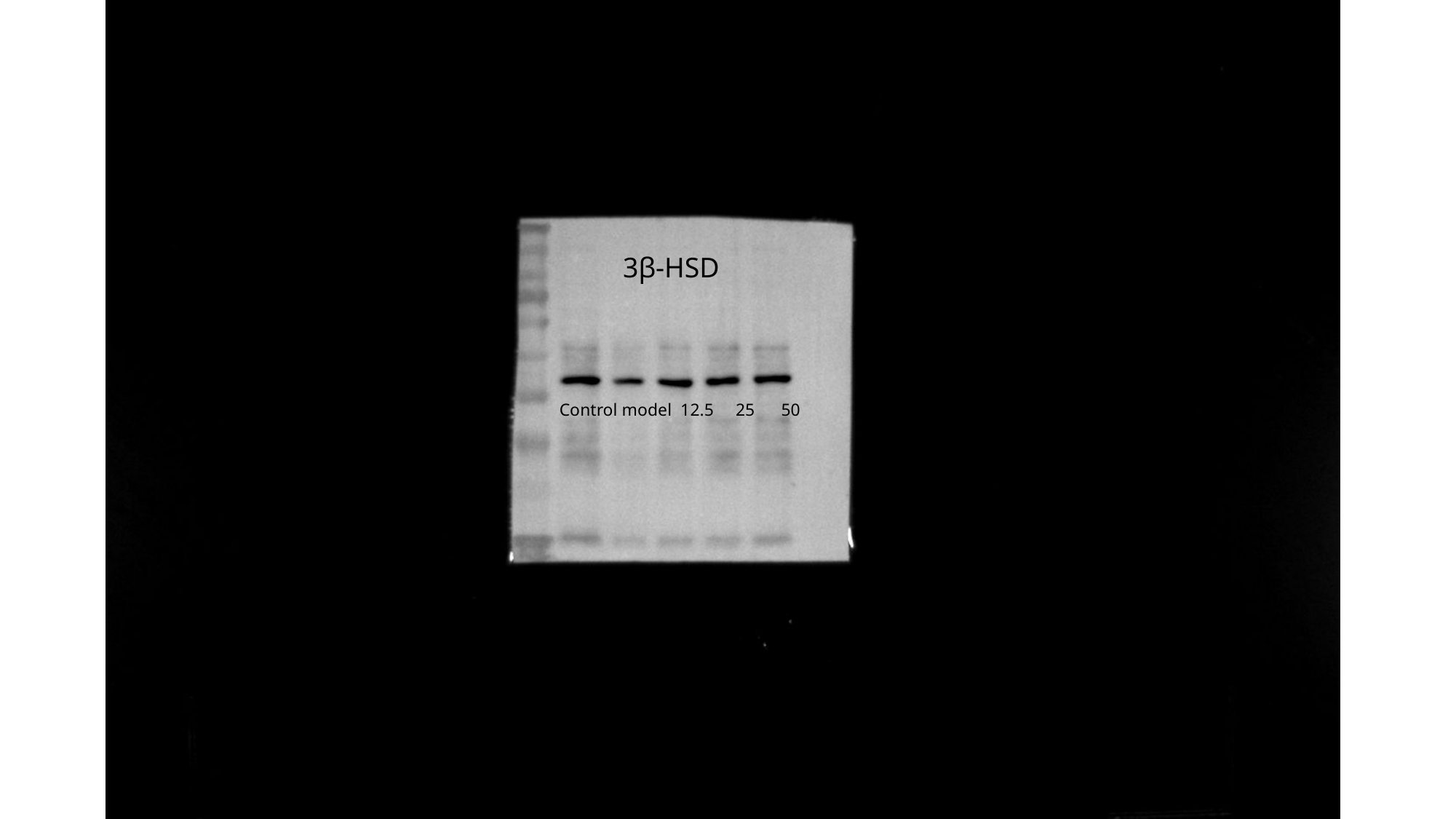

3β-HSD
Control model 12.5 25 50
